# Mapping geologic structures from Gravity and Digital Elevation Models in the Ziway-Shala Lakes basin; central Main Ethiopian rift

**DOI:** 10.1016/j.heliyon.2021.e08604

**Published:** 2021-12-14

**Authors:** Hailemichael Kebede, Abera Alemu, Dessie Nedaw

**Affiliations:** a Collage of Natural and Computational Science, Ambo University, Ambo, Ethiopia; b Computational Data Science Program, Addis Ababa University, P.O. Box 1176, Addis Ababa, Ethiopia; c School of Earth Science, Addis Ababa University, P.O. Box 1176, Addis Ababa, Ethiopia

**Keywords:** Surface and subsurface geologic structures, Lineament analysis, Gravity lineaments, Topographic lineament, Derivative filters, Upward continuation, Line module algorithm, Line density histogram

## Abstract

This study attempts to delineate subsurface lineaments for the tectonically and volcanically active region of the Ziway-Shala Lakes basin, central Main Ethiopian rift. Most of the previously mapped subsurface structures in the region under consideration focus on delineating crustal structures thicknesses and Moho depths undulations. Moreover, surface structures in the same region were mapped using analysis of Digital Elevation Model image data. On the other hand, there are few studies that have targeted in mapping geologic structures lying at depth levels between the shallower and deeper subsurface. The objective of this research is thus to map the subsurface geologic structures/lineaments to an average depth of 3 km (crystalline basement layer depth) from surface using gravity data. These investigation results are validated by Digital Elevation Model extracted lineaments. Filtering techniques including derivative filters, upward-continuation and line module algorithm of PCI Geomatica are used to extract the gravity and topographic lineaments of the region. Orientation analyses of these subsurface and surface lineaments are made using line direction histogram of the QGIS software. Accordingly, the gravity subsurface lineaments mapped in this study are found to be dominantly oriented in the NNW-SSE to NW-SE and E-W direction on average. These results appear to be contrary to the NNE-SSW to NE-SW trending surface geologic structure mapped on the bases of actual field observation carried out by previous researchers and automatically extracted lineaments based on Digital Elevation Models data considered in this research. The subsurface lineaments mapped using gravity data are believed to govern groundwater dynamics within the basin and the adjacent basins in the area. These structural lineaments which are considered to be masked in the subsurface coincide with the orientation of the Mesozoic Ogaden rift as compared to the overlying surface structures which appear to coincide with the orientation of the Cenozoic Main Ethiopian rift.

## 1 Introduction

**Figure 1:**
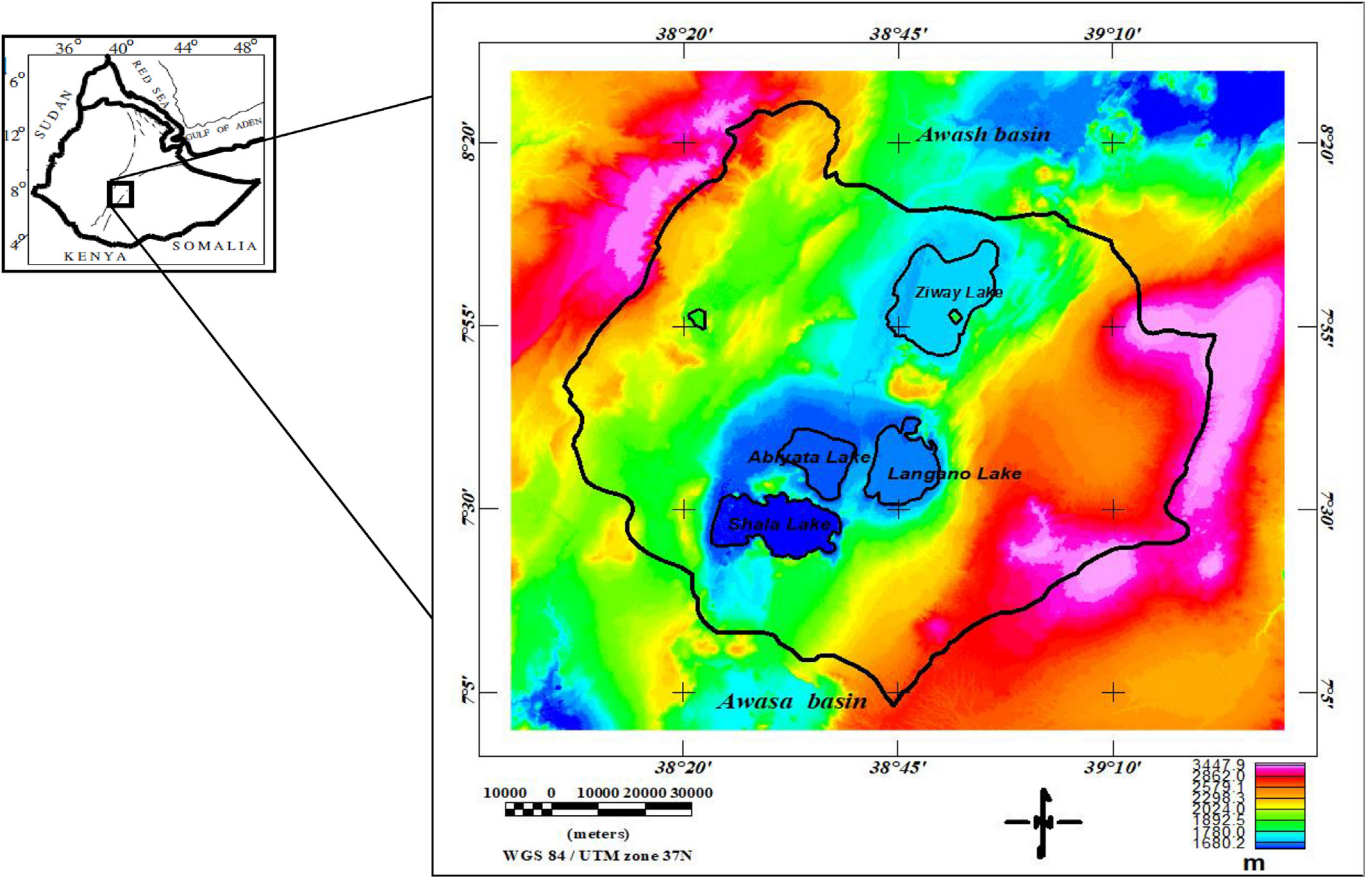
Location and topographic map (DEM) of the Ziway-Shala lakes basin and its surroundings with the main physiographic elements.

The Main Ethiopian Rift (MER) encompassing three segments, southern, central and northern MER (Woldegabriel et al., 1990; Bonini et al., 2005) is part of a bigger East African Rift system (EARS) that links the Afar triple junction and the Kenya Rift regions. The study area, Ziway-Shala Lakes basin, is located in the central part of the Main Ethiopian rift (Ayenew, 2001) and is bounded within the limits of 38^0^00′–39^0^30′E and 7^0^00′–8^0^30′N. The region is characterized by volcano-tectonic depressions having three physiographic features, the rift floor and the flanking escarpments and plateaus. The mean elevation varies from 1632 masl to 3448 masl (Figure 1).

**Figure 2:**
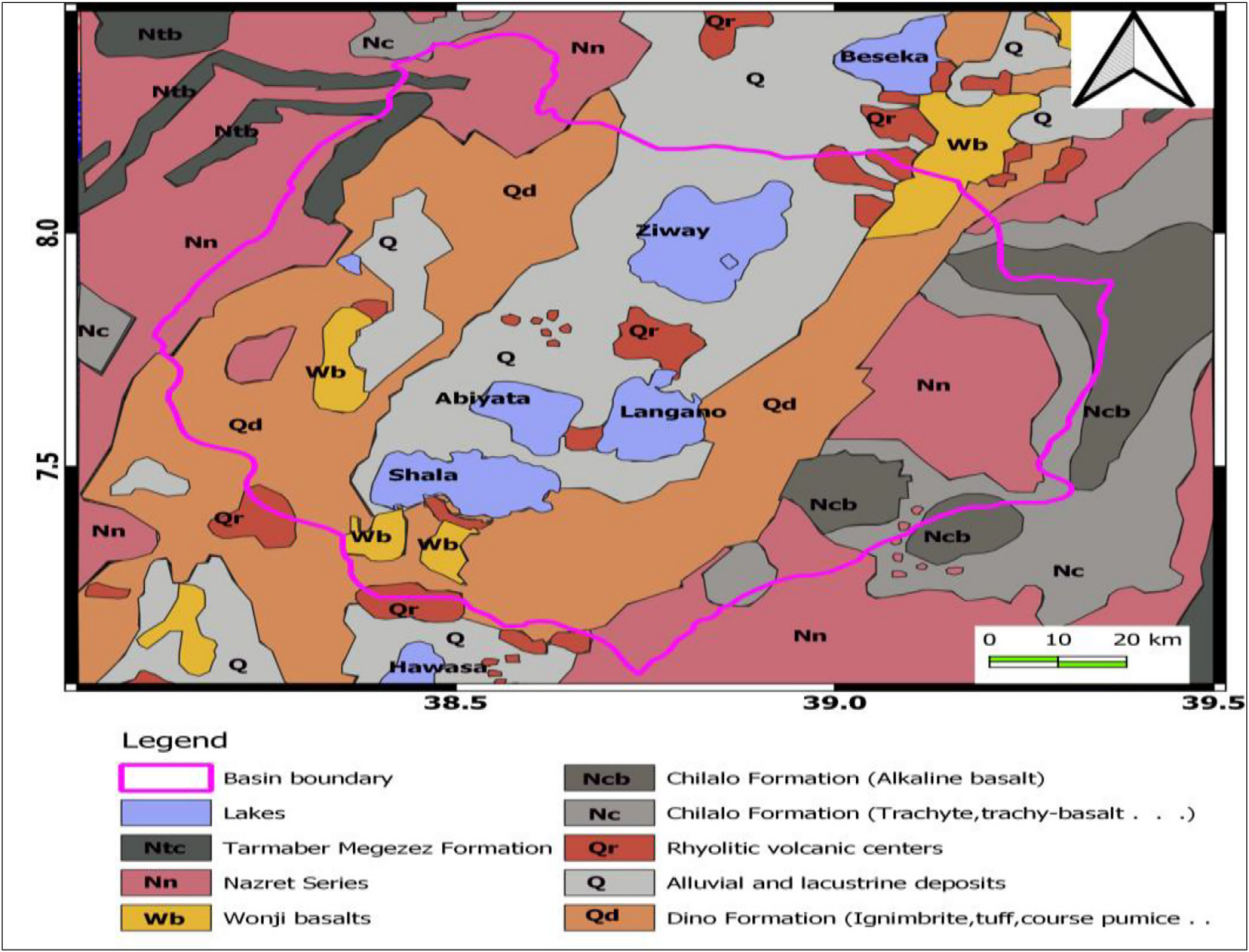
Geology of the Ziway-Shala Lakes basin, central Main Ethiopian rift modified from Tefera et al. (1996).

**Figure 3:**
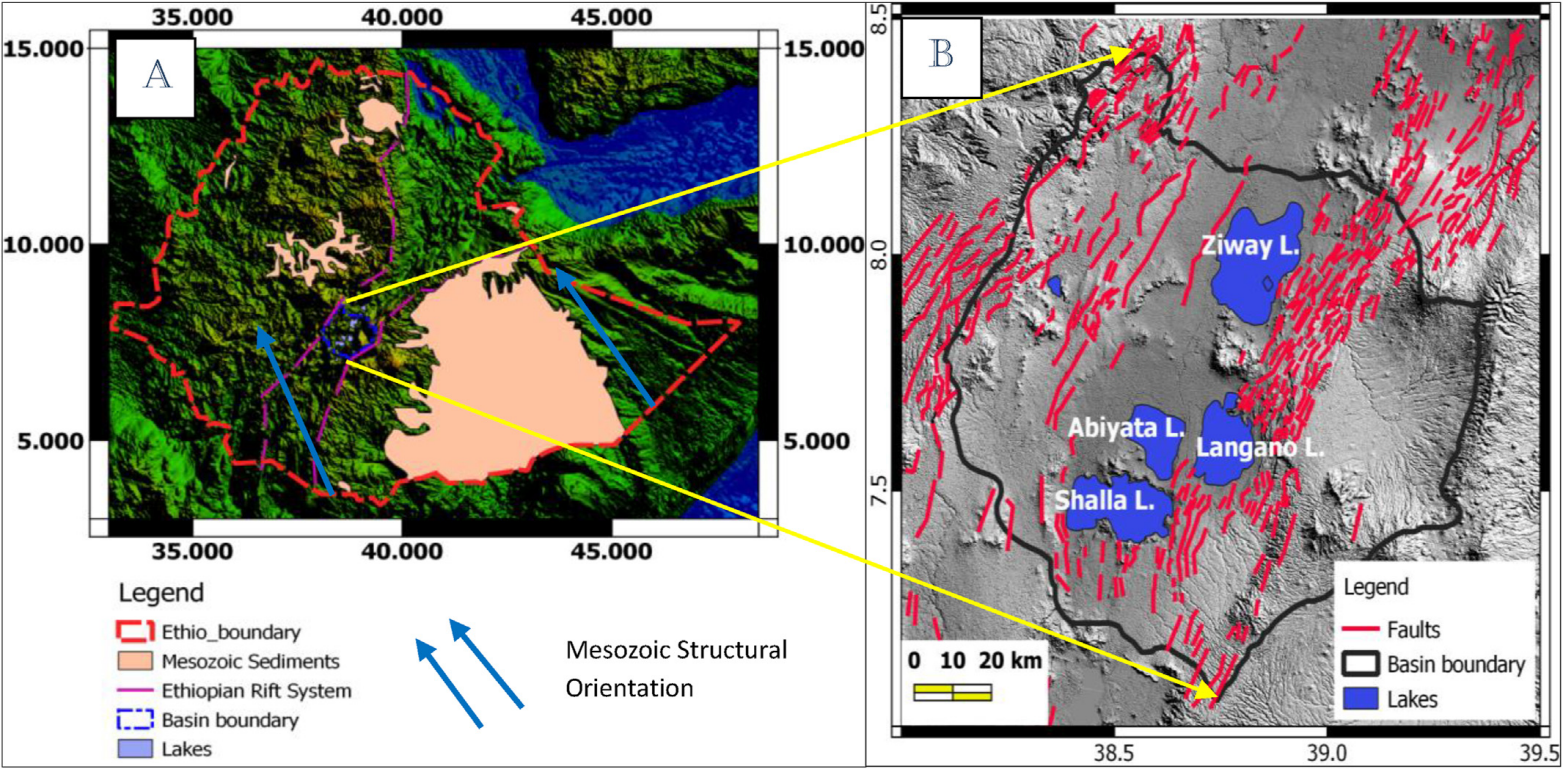
Outcropped Mezozoic structural orientation which is called pre-existing Mesozoic structures (a) structural map of the study area modified from Agostini et al. (2011) and Molin and Corti (2015) (b).

These geology and geologic structures observed in the region are due to active Cenozoic volcanic, tectonic and sedimentation processes (Abebe et al., 2007; Woldegabriel et al., 1990; Le Turdu et al., 1999). These structures are faults, joints and fractures which have surface expression as shown in the geologic map (Figure 2) and the structural map (Figure 3) of the area. These structures could constitute faults, joints and fractures with their surface expression shown in (Figures 2 and 3). These surface structures generally have N-S to NNE-SSW and NE-SW to N-S (Korme et al., 2004) orientation and are collectively called Wonji Fault Belt (WFB) (Mohor, 1962) and boundary faults (Boccaletti et al., 1998). The WFB is the youngest and most active fault system cross-cut by the pre-existing NW-SE Mesozoic Ogaden rift fault (Korme et al., 2004). These pre-existing structures have been proven to exert a significant control on the accommodation of deformation and on the distribution of strong volcanic activity (Corti et al., 2013; Abebe et al., 2007) in the region.

It is accustomed to map geological structures (lineaments) by making use of remote sensing (DEM) and geophysical data. 2D/3D modeling of potential field data can be used to map subsurface structures in various study areas (Dejene et al., 2021; Kebede et al., 2021; Toushmalani and Saibi, 2015; Mammo, 2012; Mahatsente et al., 1999). On the other hand, subsurface structures can be mapped using anomaly filtering techniques through enhancement of gravity and magnetic anomaly data, where these are automatic techniques used to detect structural lineaments (Saibi et al., 2012; Masoud and Koike, 2011; Aydogan, 2011; Gout et al., 2010; Zhang et al., 2006).

Potential field data are often represented on regular grids where equations and formulas developed for the analysis of potential field data are readily applied to gridded DEMs (Wladis, 1999). Various researchers applied filtering methods employed in potential field data to trace surface structures of an area based on DEM anomaly map (Wladis, 1999; Abdullah et al., 2010).

The geological structures in the East Africa Rift system have been described by numerous authors mainly focus on extracting the surface structures of shallow Earth origin (Molin and Corti, 2015; Agostini et al., 2011). The data used to trace these surface structures are DEM images. The surface structures in the Ziway-Shala Lakes basin mapped using DEM data and actual field observations (Agostini et al., 2011) is shown in Figure 3b. The subsurface structures of deeper origin for the same region are inferred by geophysical data (mainly gravity and seismic), most of which focus on mapping crustal structures thicknesses and Moho depth undulations.

Based on gravity data, various studies have shown that the crust thins northward along the rift (Mickus et al., 2007; Tiberi et al., 2005; Tessema and Antoine, 2004; Mahatsente et al., 1999). Refraction/wide-angle seismic reflection survey conducted along the rift (Maguire et al., 2006) support the results from gravity data. Though, its depth extent is not mentioned Korme et al. (2004) identified a pre-existing NW-SE extending Mesozoic Ogaden rift fault from gravity data. These structures cross the main Ethiopian rift in an approximately orthogonal fashion (Korme et al., 2004). In this respect, there is lack of studies that have targeted on delineating the intermediate depth (between shallower and deeper earth) geological structures at different depth levels in the Ziway-Shala Lakes basin.

By taking into consideration all the points mentioned, the objectives of this study are thus defined:

1. To map the subsurface geologic structures/lineaments to a depth of the crystalline basement (3 km) using gravity data
2. To map surface structures (topographic lineaments) from DEM data and use this information to validate (constrain) the subsurface structures mapped using the gravity data.
3. To determine the influence of subsurface lineaments (structures) on groundwater flow

In this research work we employ first vertical derivative, second vertical derivative, tilt derivative, upward continuation and line module (segment tracing) algorithms to extract information regarding surface and subsurface structures of the study area.

## 2 Datasets and methodology

### 2.1 Gravity and Digital Elevation Model data

**Figure 4:**
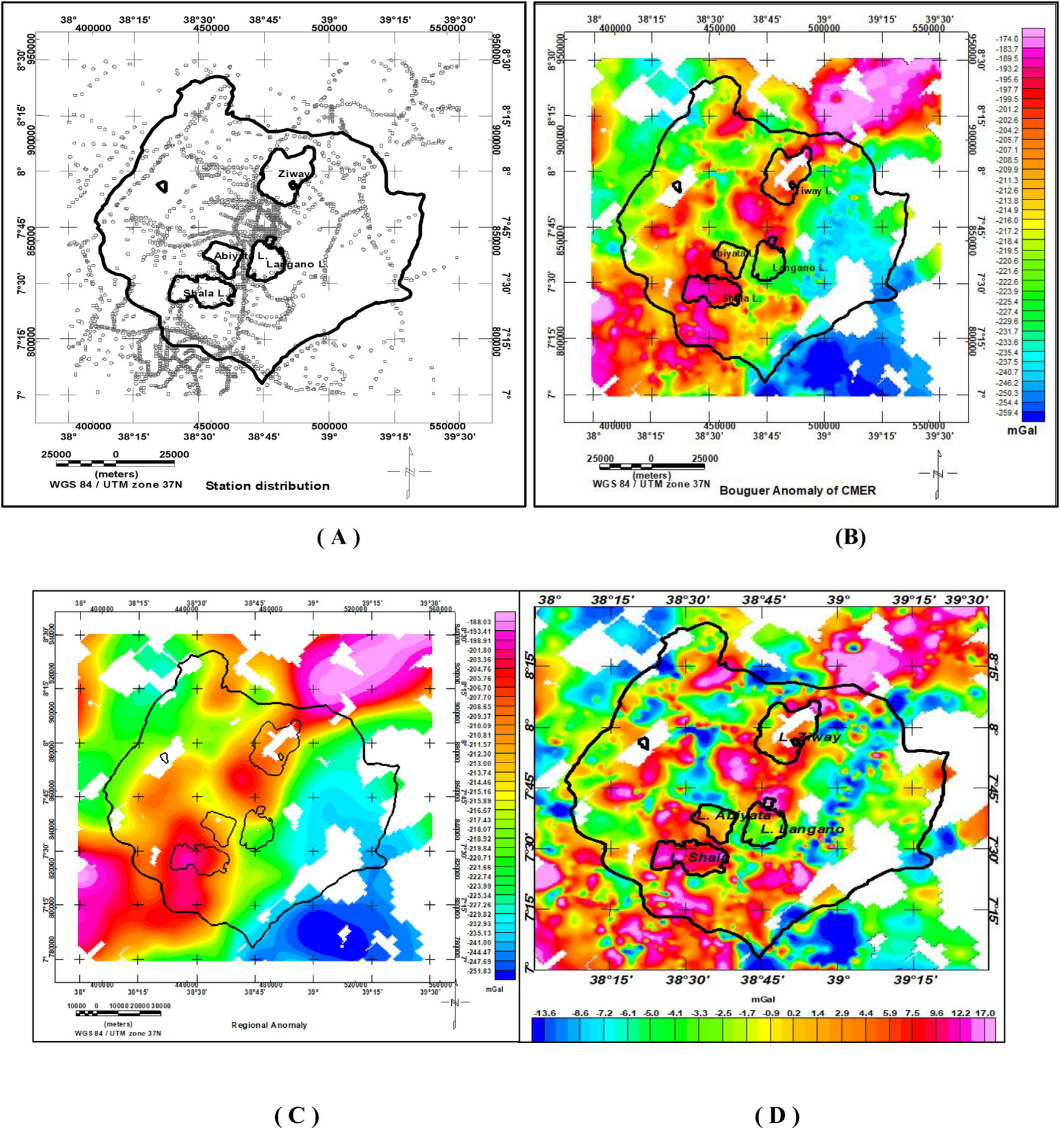
Gravity stations distribution map (A) Bouguer anomaly map (B) regional anomaly map (C) and residual anomaly map (D).

Gravity and Digital Elevation Model data sets are examined for subsurface and surface structures beneath the Ziway-Shala Lakes basin, central Main Ethiopian Rift. The data acquisition and processing are documented as follows:

Ground based gravity data were obtained from Geological Survey of Ethiopia and PhD thesis work (Alemu, 1992). This data were reprocessed and homogenized to the International Gravity Standardization Network 1971 (IGSN71). The 1967 international gravity formula, a reduction density of 2.67 g/cm^3^ and sea level as a datum are used. The computed complete Bouguer gravity anomaly values are gridded to generate the complete Bouguer anomaly map (Figure 4(b)) of the study area. The regional gravity anomaly is estimated using upward continuation filter with an upward continuation height of 6 km (Kebede et al., 2020; Figure 4(c)). The residual gravity anomaly map (Figure 4(d)) of the region is then compiled by subtracting the estimated regional from the observed complete Bouguer gravity anomaly.

A DEM data an Advanced Space-borne Thermal Emission and Reflection Radiometer (ASTER) gridded imagery data used to represent elevation information of the study area from which surface geological structures are mapped from. The DEM data employed here have a 30 m spatial resolution (Figure 1).

According to Wladis (1999) since DEM data is a gridded data, grid-based interpretation methods used in analysis of potential field data can be used to extract surface lineaments for a region of interest.

### 2.2 Methodology used to extract geological structures

Mapping surface and subsurface structures based on DEM and potential field data is a well-practiced and established procedure. Contacts between rocks that have different physical properties usually occur along lineaments which consist of faults fractures, etc. Such lineaments which could show major subsurface structures are extracted using image filtering algorithms applied on gravity anomaly data (Aydogan, 2011; Saibi et al., 2008). Topographic lineaments (Kassou et al., 2012; Abdullah et al., 2010; Jordan et al., 2005; Wladis, 1999) are traced from DEM data using the same filtering techniques used in the analysis of gravity data. The governing mathematical equations for the filter types considered are described below.

#### 2.2.1 First and second vertical derivative

Vertical derivatives (VDR) are data filtering techniques used for the enhancement of the shallow gravity source features (Wladis, 1999). Gridded gravity and DEM anomaly data input to VDR filters can be expressed as a function in Cartesian co-ordinate system denoted by F=f(x,y,z).

The function which shows the change of field/elevation with respect to depth (z) is expressed as first vertical derivative (FVDR) (Eq. (1)):

(1) $$FVDR=-\frac{\partial f}{\partial z}$$

and second vertical derivative (SVDR) (Eq. (2)):

(2) $$SVDR=-\frac{\partial^{2}f}{\partial z^{2}}$$

The Oasis montaj Geosoft standard software (“Geosoft Oasis montaj version 7.0,” n.d.) is used to generate the first and second order derivatives of the gridded DEM image. The procedures have effects of enhancing localized shallow (near surface) sources and generate lineaments.

The tilt derivative (θ) of gravity anomaly, **F**, is expressed as a ratio of its first vertical derivative to total horizontal derivative (Verduzco et al., 2004) (Eq. (3)):

(3) $$\theta =TDR=tan^{-1}\frac{\frac{\partial F}{\partial \text{z}}}{\sqrt{{\left(\frac{\partial F}{\partial x}\right)}^{2}+{\left(\frac{\partial F}{\partial y}\right)}^{2}}}$$

Where, $\frac{\partial F}{\partial x},\;\frac{\partial F}{\partial y}$ and $\frac{\partial F}{\partial \text{z}}$ are the derivatives of the gravity anomaly, **F,** with respect to x,yandz directions.

A mathematical property of arctan restricts the value of θ to lie between $-\frac{\text{π}}{2}\text{ ​and ​}-\frac{\text{π}}{2}$ or between $-90^{0}\text{ ​and ​}90^{0}$.

The filter enhances and sharpens the anomalies with zero value contours (zero crossing) which indicate lithological/structural contacts.

#### 2.2.2 Upward continuation

Vertical derivative and tilt derivative filters generally enhance effect of the shallower earth but not necessarily effect of the deeper earth. The regional anomaly resulting from the deeper earth is approximated using the upward continuation filter which is mathematically expressed by Gupta and Ramani (1980) and Jacobsen (1987) (Eq. (4)) as:

(4) $$H_{reg}\left(k\right)=S_{0}\left(k\right)\;e^{-2\pi kz_{0}}$$

Where $S_{0}\left(k\right)$ is Bouguer anomaly, k is the wave number and $z_{0}$ is the continuation height.

The deeper gravity source signatures are isolated by upward continuing the observed Bouguer anomalies to a higher elevation. According to Jacobsen (1987), if a potential field is upward continued to a certain height, Z, then it will map sources situated at and below the depth Z/2. The residual anomaly is then obtained through subtraction of this regional anomaly from the observed Bouguer anomalies.

**Figure 5:**
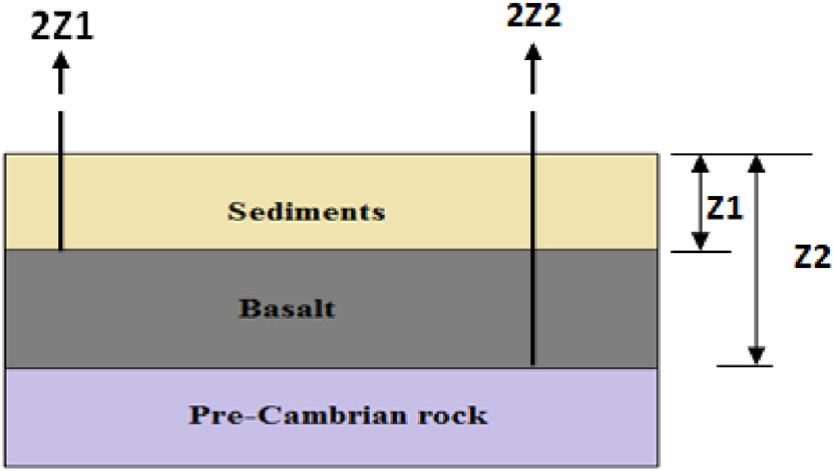
Schematic representation of three earth layers for extraction of the gravity field anomaly response of a slab (Example, basaltic rock formation layer) located between depths Z_1_ and Z_2_.

Jacobsen (1987) also showed that the field generated by a slab located at depths in between Z_1_ and Z_2_ is simply the difference between the fields resulting from upward continued heights of $2{\text{Z}}_{1}\;and\;2{\text{Z}}_{2}$ (Figure 5).

**Figure 6:**
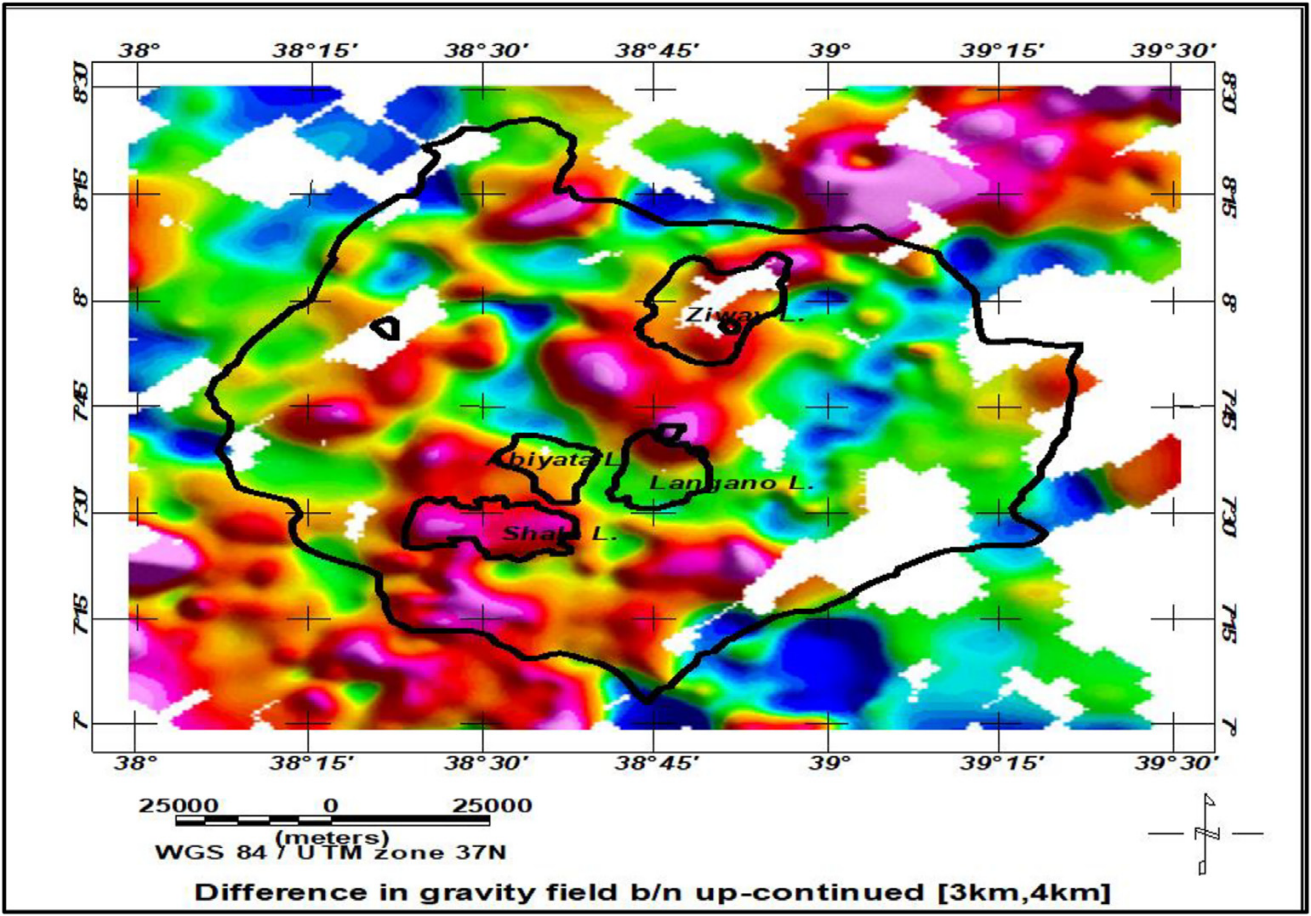
The gravity anomaly maps of sources (sliced slabs) compiled by taking the differences in up-continued gravity field between 3 km and 4 km.

The following procedures are followed for the extraction of lineaments emanating from a sandwiched (sliced) gravity source distribution (Figure 6).

✓ Upward continuation of the observed Bouguer anomaly to a heights of 0.5, 1, 2, 3, 4, 5 and 6 km.
✓ Obtaining differences of consequently upward continued anomalies to generate anomalies originating from slabs (slices) located at consecutive depths between 0.25 & 0.5, 0.5 and 1, 1 and 1.5, 1.5 and 2, 2 and 2.5, 2.5 and 3.0, 1.5 and 3 km.
✓ For anomalies resulting from each slice, line module algorithm of PCI Geomatica and tilt derivative filters are applied to extract lineaments resulting from each slice.

The Bouguer gravity anomaly map (Figure 4(b)) is upward continued to heights of 0.5, 1, 2, 3, 4, 5 and 6 km in order to image sources buried at depths of 0.25, 0.5, 1, 1.5, 2, 2.5 and 3.0 km respectively. This upward continuation filter (low-pass filter) generates regional anomalies subtracted from each other giving rise to regional anomalies generated by slabs (sliced slabs) located at depths between 0.25 & 0.5, 0.5 & 1, 1 & 1.5, 1.5 & 2, 2 & 2.5, 2.5 & 3.0 and 1.5 & 3 km. As an illustration, the anomalies generated by sliced slabs between the depths 1.5 and 2 km is depicted in Figure 6.

Oasis Montaj Geosoft is used to filter the regional anomalies generated by sliced slabs located at the depths considered and PCI Geomatica software is used to extract the geologic lineaments occurring in the area to a depth of 3 km (≈mean crystalline basement depth) (Kebede et al., 2020). The differenced regional anomalies (anomalies of the sliced slabs) are exported as shaded-relief Geotiff 256 Grey (8 bit) images to be used as an input to the Line module algorithm of PCI Geomatica V10. The exported images emphasize gradients in anomaly grids and are useful for displaying strong linear features observed in the images. The methods automatically identify lineation in three steps including edge detection, thresholding and curve extraction (details given in section 2.2.3).

#### 2.2.3 Line module algorithm

The LINE option of PCI Geomatica software extracts lineaments automatically from images and records the polylines in a vector segment (Abdullah et al., 2010). This algorithm is designed to extract linear and curvilinear features from radar images or from optical images.

For mapping reasonably acceptable lineaments, the images should be enhanced with different filtering techniques which may include shaded-relief methods performed using ArcGIS 10.3 software or principal component analysis (PCA) method performed using Image processing software such as ENVI 5.1. The PCA is a statistical technique which removes data redundancy and isolates noises by enhancing images which could finally be used as an input to the filters for extracting geological lineaments (Adiri et al., 2016).

The other image enhancement method is the shaded-relief image techniques which generate a pan sharpened 8 bit gray scale reflected bands to be used as input to Line module of PCI Geomatica V10 software to automatically extract geological lineaments. This algorithm detects the lineation in three steps which include edge detection step, thresholding step and curve extraction step. The input output parameters pertaining to this algorithm including their relationship can be found in the website http://www.pcigeomatics.com/geomatica-help/references/pciFunction_r/python/P_line.html.

The optimal choice of the input/output parameters is chosen by a trial and error process with the shape and density of the generated lineaments taken in to consideration. The default input parameters used by PCI Geomatica algorithm including the selections made in this research are listed in Table 1.

**Table 1:** The different parameters input to line module PCI Geomatica software to automatically extract lineaments in Ziway-Shala Lakes Basin.

No.	Parameters	Parameters value option	Default (Choice 0)	Choice 1	Choice 2	Choice 3
1	Radius of filter in pixels (RADI)	5, 10, 20, 50, 100	10	10	5	5
2	Threshold for edge gradient (GTHR)	5, 10, 20, 50, 100, 200, 255	100	20	20	30
3	Threshold for curve length (LTHR)	5, 10, 20, 50, 100	30	30	20	10
4	Threshold for line fitting error (FTHR)	1, 3, 5, 7, 9, 10, 20, 50, 100	3	3	3	3
5	Threshold for angular difference (ATHR)	0, 15, 30, 45, 60, 75, 90	30	30	15	15
6	Threshold for linking distance (DTHR)	5, 10, 20, 50, 100	20	20	10	10

Mapping geological structures (lineaments) of intermediate depth in the region considered are performed using different software such as Geosoft, ENVI 5.1, PCI Geomatica V10, ArcGIS 10.3 and QGIS.

## 3 Results and discussion

Geological structures such as faults, fractures and joints can be extracted from analysis of gravity and DEM data. The application of different filtering algorithms on these anomalies/images generates gravity and topographic lineaments outlined here below.

### 3.1 Subsurface lineaments extraction using gravity anomalies

**Figure 7:**
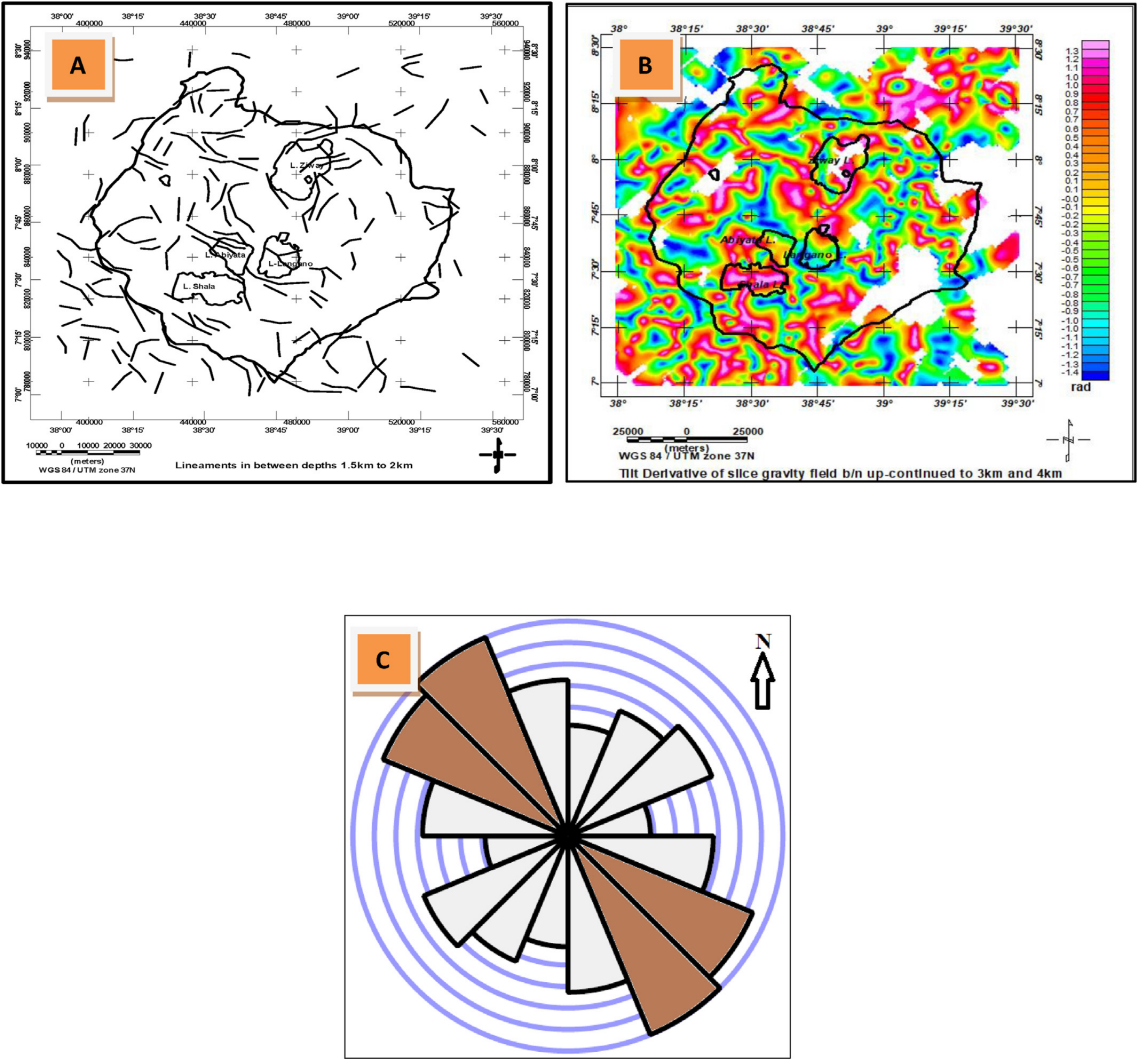
Sample lineament maps (a) for depths between 1.5 and 2.0 compared with lineament map generated using tilt derivative (b) and rose diagram plot showing orientations of the subsurface lineaments (c).

Figure 7 reveals sample gravity lineaments extracted in the study area based on the methodologies mentioned in sections 3.2, 3.3 and 3.4. These includes lineaments extracted based on Line module algorithm (Figure 7(a), tilt derivative techniques (Figure 7(b) and rose diagram plot showing the overall subsurface lineaments orientation constructed based on line direction histogram module of QGIS (Figure 7(c)).

The major geological structures (lineaments) (Figure 7) which are seen in the form of linear geometries are extracted through analyzing gravity data. The line module algorithm of PCI Geomatica is used to extract these lineaments (Figure 7(a)). These lineaments are compared with lineaments mapped using the tilt derivative method (Figure 7(b)). Their comparison shows that both methods give similar results in identifying the location, orientation and density of lineaments in the study area. The extracted lineaments are dominantly oriented NNW-SSE to NW-SE and E-W (Figure 7(c)) which thought to coincide with the direction of pre-existing Mesozoic structures previously identified in the area (Korme et al., 2004). The result also shows lineaments trending NE-SW (Figure 7(c)) that coincides with the orientation of the quaternary faults of the Main Ethiopian Rift system that comprises the study area.

**Figure 8:**
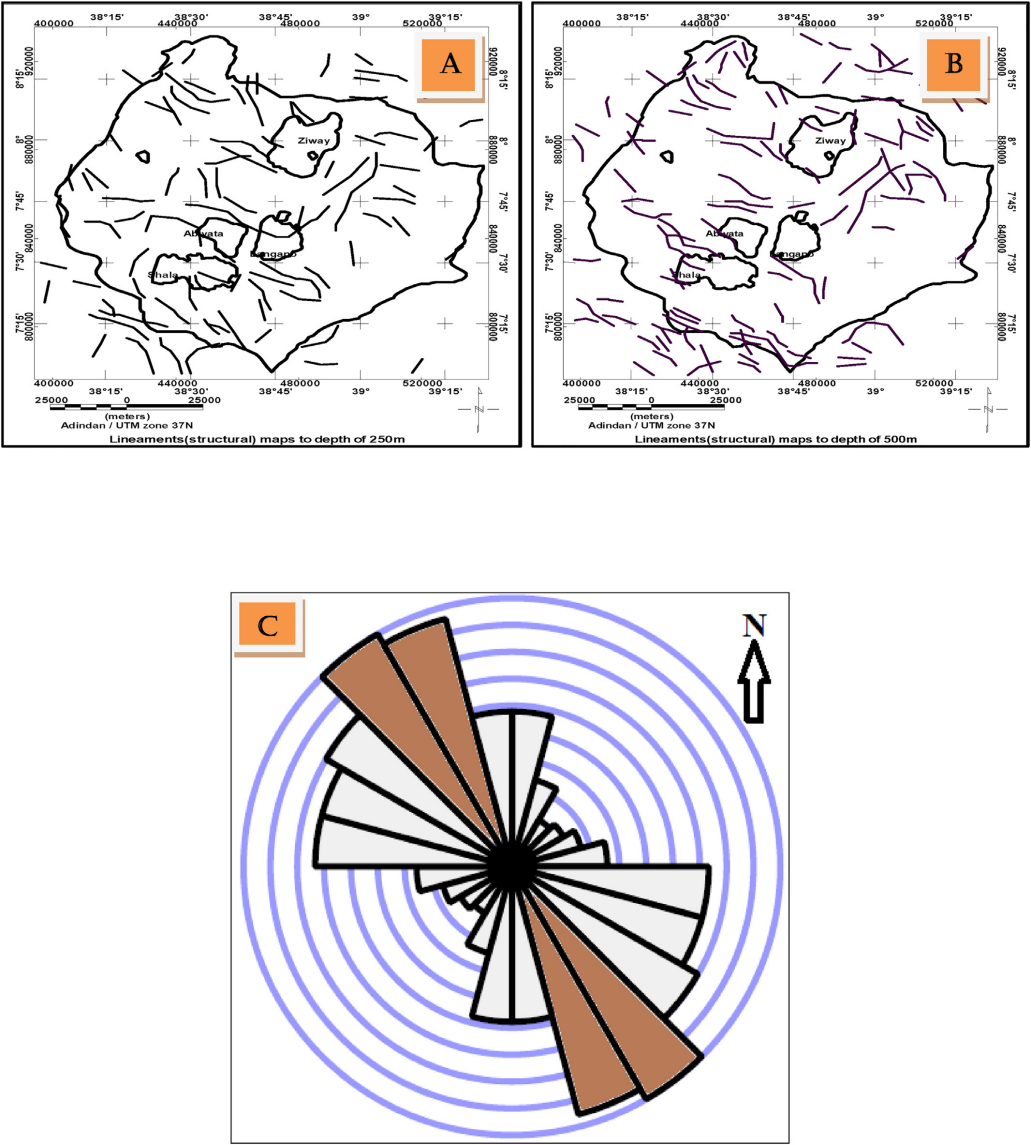
Sample lineaments maps extracted from the residual gravity anomaly map to depths of 0.25 km (a), depth of 0.50 km(b) and line density rose diagram plot showing orientations of the subsurface lineaments (c).

The subsurface lineaments can also be extracted from residual gravity anomalies at different depth levels. The estimated regional anomalies generated using upward continuation to heights of 0.5, 1, 2, 3, 4, 5 and 6 km are subtracted from observed Bouguer anomaly to extract residual anomalies caused by sources extending to depths of 0.25 km, 0.5 km, 1.0 km, 1.5 km, 2.5 km and 3.0 km respectively. These residual anomalies are then converted to 8 bit shaded relief images to be used as an input to line module algorithm which help to extract subsurface lineaments at different depth levels. The sample identified lineaments (Figure 8(a), (b)) are dominantly oriented in a NW-SE direction as also revealed by the rose diagram plot (Figure 8(c)). These linear features (lineaments) are in agreement with respect to their location, orientation and density with those lineaments extracted based on the regional gravity anomalies caused by sliced slabs (Figure 6).

### 3.2 Surface lineaments extracted from DEM

The topographic lineaments considered in this section are traced using the procedure and methods outlined in sections 2.2.1 and 2.2.4.

#### 3.2.1 First and second vertical derivative

**Figure 9:**
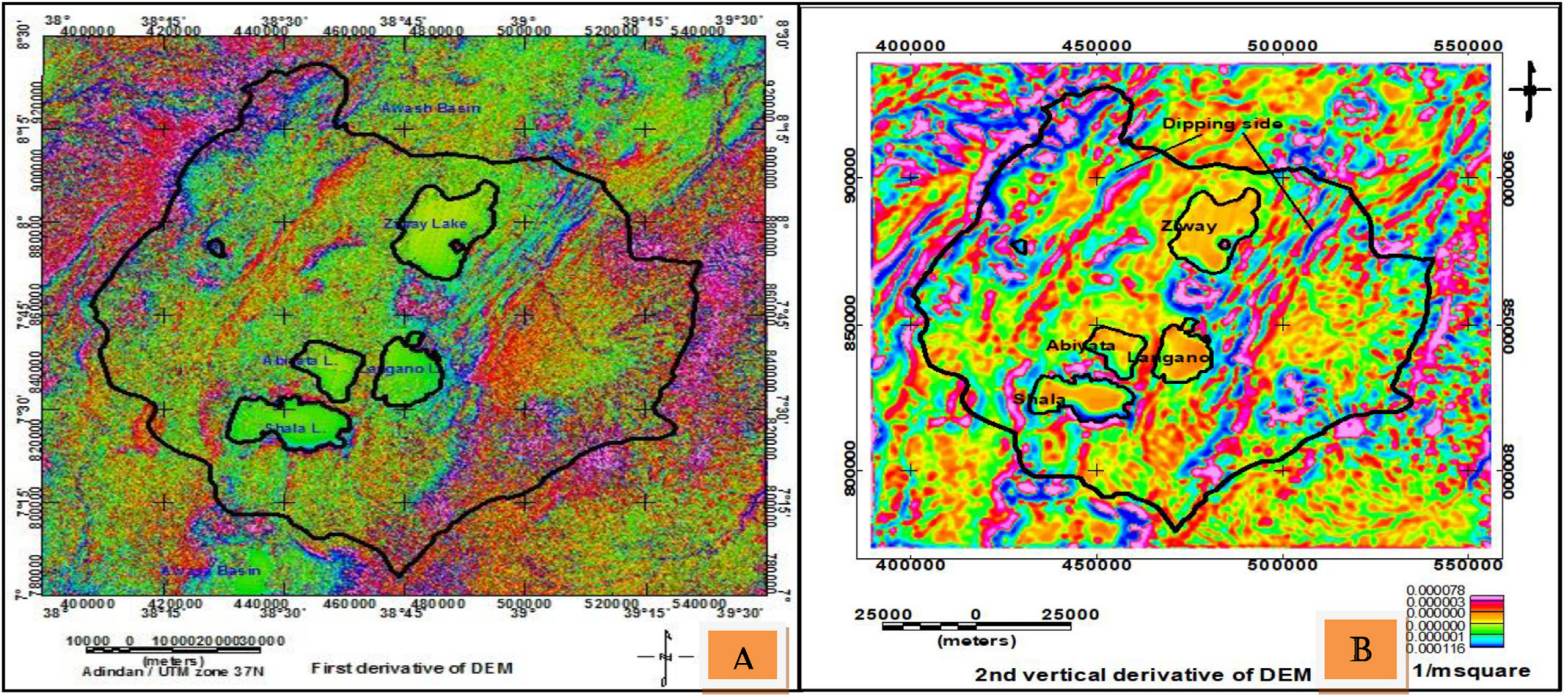
First vertical derivatives of topographic (DEM) data (a) Lineaments extracted from DEM using second order derivative with dip directions towards low color contrast (e.g., blue color) (b).

The application of first vertical derivative filter on DEM image map generates slope image map shown in Figure 9(a). This map reveals surface structures coinciding with the existing Cenozoic fault patterns observed in the study area (Agostini et al., 2011). Similarly, according to Wladis (1999) the second order derivative filter was used for detection of lineaments. This method has the effect of enhancing anomalies over anomalous sources. The topographic lineaments (Figure 9(b)) mapped using this method also shows the dip directions of the structure towards blue color contrast.

The lineaments extracted using derivative filters (Figure 9) give clearer picture of shallow source anomalies with the linear features indicating geologic structures observed in the area. Furthermore, the linear topographic lows may be thought to indicate depressions existing in the area.

#### 3.2.2 Line module of PCI Geomatica

**Figure 10:**
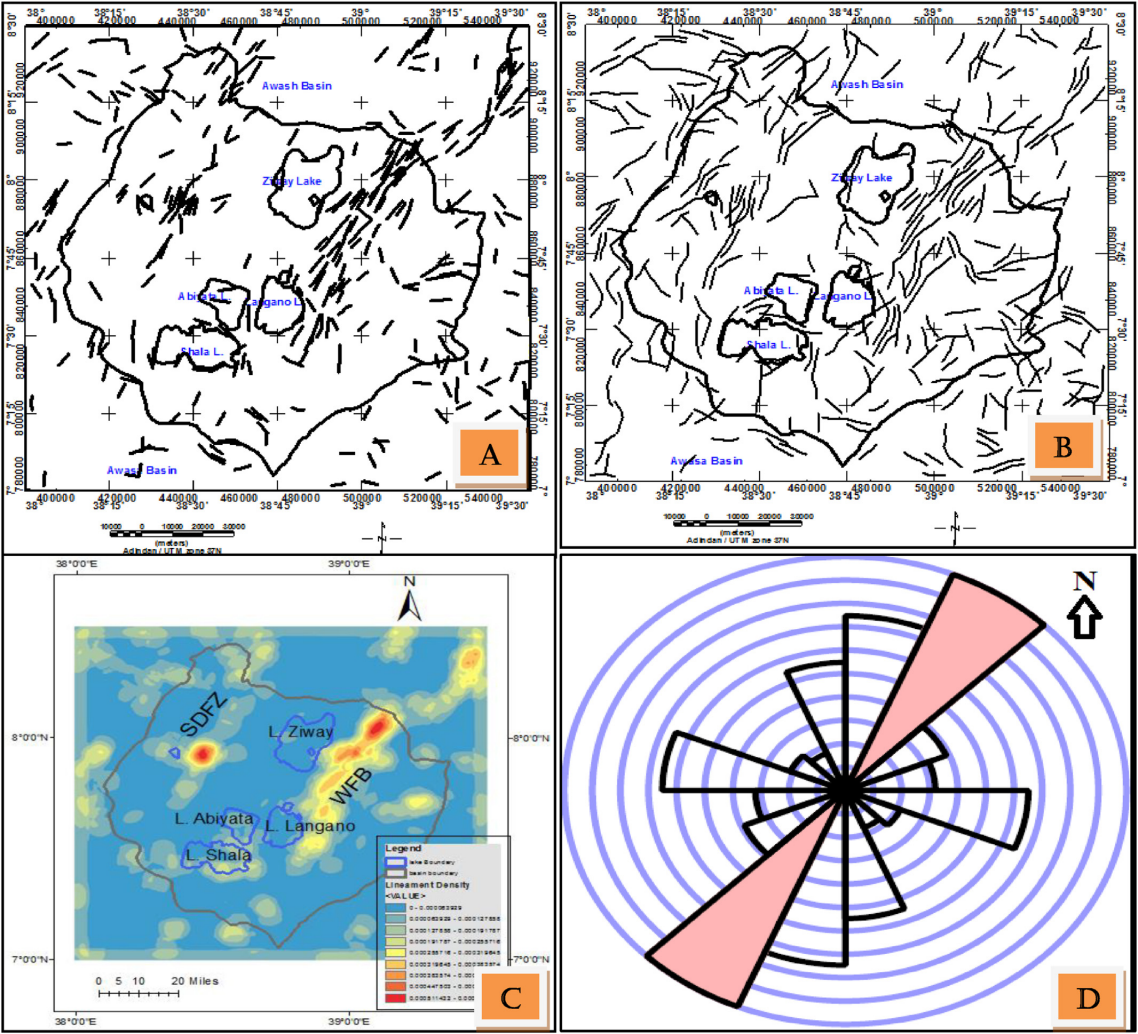
Automatically extracted lineaments with DEM slope gradient as an input with parameters taken from choice 1 (a); choice 2(b) and lineament density map of the study area (c) using lineaments shape file from lineaments (a) as an input. Rose diagrams showing the overall orientation (directional trend) of surface lineaments extracted from DEM (d).

Lineaments are automatically extracted using the Line module algorithm with enhanced slope image of DEM and the input parameters options as choice 1 and choice 2 indicated in Table 1 resulting in Figure 10(a) and (b). A lineament density map (Figure 10(c)) is derived from the slope image lineament map (Figure 10(a)) fed as an input to ArcGIS software.

With the default parameters (choice 0) (Table 1) in PCI Geomatica software few lineaments (faults) were mapped in the area. However, with a change of threshold edge gradient from 100 to 20 (choice 1) and all the others parameters kept constant, the program generates the lineaments shown in Figure 10(b).

Similarly, the result based on input parameters given in choice 2 produces lineaments shown in the Figure 10(a). These structures are all similar in orientation and location to that of lineaments traced based on choice 1. However, they are more linear in shape and shorter in size. In this case all the curved structures are wiped-out with their linearity preserved. Generally, the two parameter options chosen mostly generate lineaments of the study area. However, there is a need to experiment on the selection of the input parameters for better extraction of lineaments in the study area. The lineation density map (Figure 10(c)) shows more lineaments on the Eastern escarpment where the WFB is located as compared to those on the Western escarpment where SDFZ is located including their accompanying border faults. The extracted geological structure (lineaments) statistically analyzed (trend analyzed) and plotted in the form of rose diagram (Figure 10(d)).

**Figure 11:**
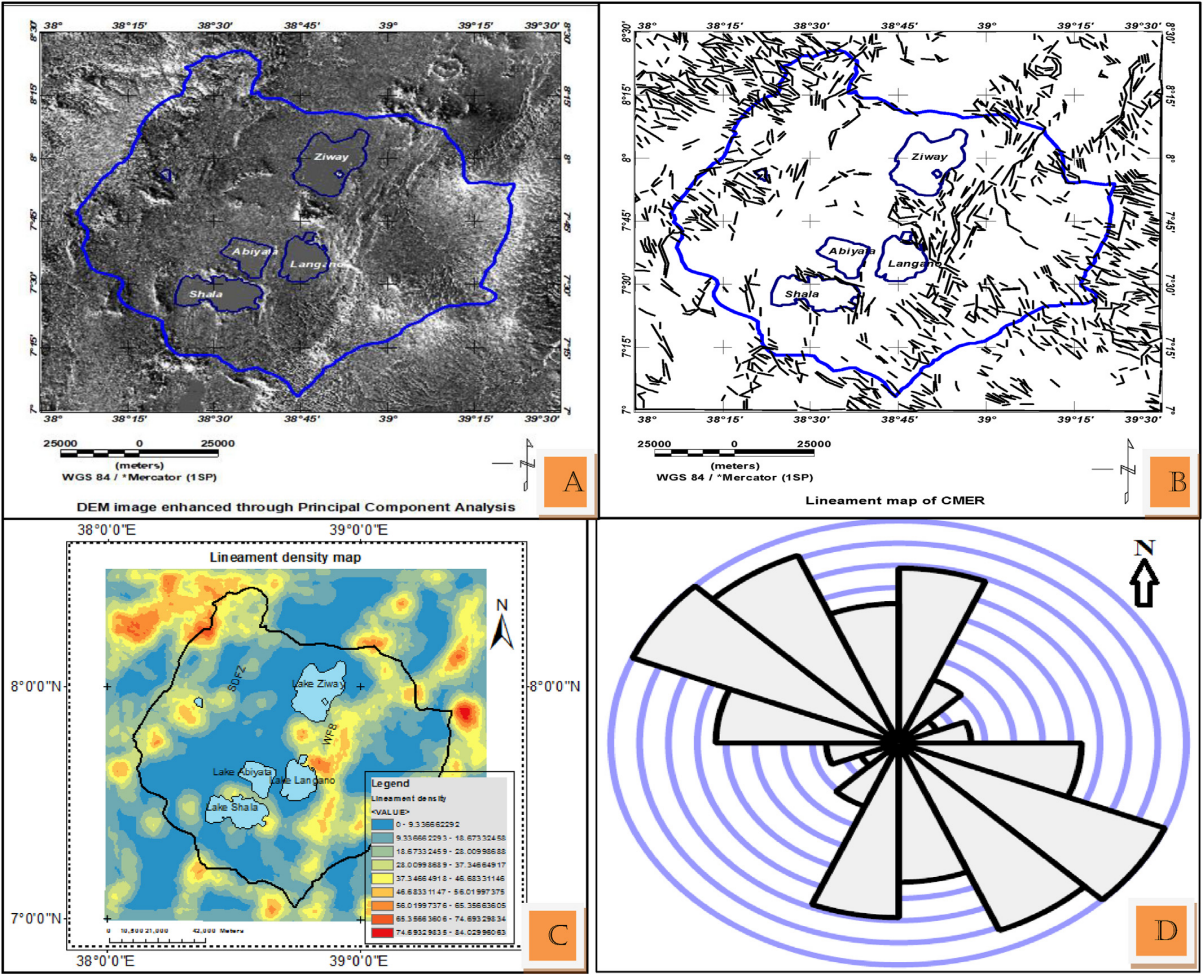
Enhanced DEM map using Principal Component Analysis (a) automatically extracted lineaments from DEM using PCI Geomatica software with image (a) as an input (b) Lineaments generated in (b) was exported to ArcGIS 10.3 where all processing and density map is generated and shown in (c) the Rose diagram showing dominant NW-SE and less dominant NNE-SSW trending lineaments.

The lineaments generated with PCA enhanced DEM image (Figure 11(a)) input to the line module of Geomatica software is shown in Figure 11(b). The result shows that, the mapped structures agree with previously identified fault maps in location, orientation and density. However, in this work more lineaments were mapped. Higher densities of lineaments are observed at WFB and SDFZ and lower density of lineaments corresponding to the sedimentary units of the rift floor Figure 11(b).

In summary, the lineaments extracted with first derivative of DEM as an input to Line module PCI Geomatica (Figure 10(a)) mostly agree with fault map (Figure 2(b)) previously mapped in the area. Most of these lineaments oriented NNE-SSW as the summery made by line density rose diagram shows (Figure 10(d)). PCA enhanced DEM input to Line module algorithm of PCI Geomatica (Figure 11(b)) equivalently map the structure with more new lineaments. In both lineament extraction procedures it is observe that too many lineaments than the true faults or tectonic features of the study area.

Most of the deep seated lineaments extracted from gravity data oriented NNW-SSE to NW-SE (Figures 7 and 8). Few of these lineaments traced using these data trends N-S and NE-SW (Figures 7(c) and 8(c)). Majority of the traced lineaments from DEM image trends NNE-SSW to NE-SW and N-S (Figure 10(d)) direction which agree with few gravity lineaments in the study area (Figures 7(c) and 8(c)). This shows few surface lineaments continued down depth. Minor surface lineaments trending along NW-SE coincide with the orientation of most subsurface lineaments extracted using gravity data.

Furthermore, most surface and subsurface lineaments out of the Main rift axis in an Ethiopian plateau oriented in the direction of pre existing structural orientation (NW-SE) (Figure 11(d)). This was also reveled by different researchers that the crust outside the rift axis in Ethiopian Plateau has not been modified significantly by Cenozoic rifting and magmatism (Dugda et al., 2005; Gani et al., 2008).

## 4 Hydro-geological significance of the mapped lineaments

The study region enclose hydrologically closed Ziway–Shala lakes basin (Le Turdu et al., 1999; Chernet et al., 2001). Observation shows that there is no evidence of significant groundwater outflow from this basin (Legesse et al., 2004). Isotopic evidence (Darling et al., 1996) and groundwater flow modeling (Ayenew, 2001) showed the ground water flow from southern Awasa basin towards the low-lying and deep Ziway-Shala lakes basin. These should increase the water resource in the basin. However, the water in the basin is declining from time to time.

Using hydrological data taken from (Ayenew, 2002), the computation of the water balance of Ziway-Shala lakes basin was conducted and showed positive value. These lead the conclusion that there should be a structural unit that conducts groundwater outflow from this basin and among the inter-basins in the region. Therefore, the subsurface structures mapped at different depth levels in this study and those lineaments crossing the water divide believed to govern the groundwater outflow from Ziway-Shala lakes basin. These finding also support the structural control of the groundwater dynamics identified (Kebede et al., 2021).

## 5 Conclusion

One way of studying the geological structure of an area is through studying linear features (lineaments) which could be extracted from gridded data anomalies. In this paper gravity and Digital Elevation Model (DEMs) anomaly data are used to map the corresponding gravity and topographic lineaments of the study area. The first and second vertical derivatives; tilt derivative, upward continuation, line module algorithms are used to automatically extract lineaments in the study area. Most subsurface lineaments extracted from gravity data oriented NNW-SSE to NW-SE directions which are against most surface structural orientation (NNE-SSW to NE-SW) mapped earlier by different researchers and extracted automatically based on DEM data considered in this research. The subsurface lineaments orientation might be due to the pre-existing subsurface structures crossing the rift orthogonally while surface structures might be due to Cenozoic rifting activities. A higher surface lineament density is observed in the eastern parts of the study area than the western side. Out of the rift most of the surface lineaments are oriented NW-SE which coincides with gravity data extracted pre-existing structures that strike the MER orthogonally. It can be concluded from the result that the integration of extracted topographic lineaments (surface structures) with potential field lineaments (subsurface structures) will add some information on the enhancements of the previously extracted structural map of the area. The identified structural lineaments are believed to govern the groundwater dynamics in the Ziway-Shala Lakes basin and among the adjacent inter-basins.

## Declarations

### Author contribution statement

Hailemichael Kebede: Conceived and designed the experiments; Performed the experiments; Analyzed and interpreted the data; Contributed reagents, materials, analysis tools or data; Wrote the paper.

Abera Alemu & Dessie Nedaw: Analyzed and interpreted the data; Contributed reagents, materials, analysis tools or data; Wrote the paper.

### Funding statement

This research did not receive any specific grant from funding agencies in the public, commercial, or not-for-profit sectors.

### Data availability statement

Data will be made available on request.

### Declaration of interests statement

The authors declare no conflict of interest.

### Additional information

No additional information is available for this paper.
